# Circulating RNA Molecules as Biomarkers in Liver Disease

**DOI:** 10.3390/ijms151017644

**Published:** 2014-09-30

**Authors:** Liviu S. Enache, Elena L. Enache, Christophe Ramière, Olivier Diaz, Ligia Bancu, Anca Sin, Patrice André

**Affiliations:** 1 Université de Lyon, Université Lyon 1, Lyon F-69008, France; E-Mails: christophe.ramiere@inserm.fr (C.R.); olivier.diaz@inserm.fr (O.D.); patrice.andre@inserm.fr (P.A.); 2 University of Medicine and Pharmacy Tirgu Mures, 38 Gh. Marinescu st., Tirgu Mures 540142, Romania; E-Mails: dincaluminita@yahoo.com (E.L.E.); ligiabancu@yahoo.com (L.B.); anka_sinn@yahoo.com (A.S.); 3 Emergency County Clinical Hospital, 50 Gh. Marinescu st., Tirgu Mures 540136, Romania; 4 Inserm U1111, 21 Avenue Tony Garnier, Lyon F-69007, France; 5 CIRI, Centre International de Recherche en Infectiologie, Université de Lyon, 21 Avenue Tony Garnier, 69365 Lyon Cedex 07, France; 6 Ecole Normale Supérieure de Lyon, 15 parvis René Descartes, BP 7000 69342 Lyon Cedex 07, France; 7 CNRS, UMR5308, 21 avenue Tony Garnier, 69365 Lyon Cedex 07, France; 8 Hospices Civils de Lyon, Hôpital de la Croix Rousse, Laboratoire de Virologie, Lyon F-69004, France

**Keywords:** liver disease, biomarker, cell-free RNA, miRNA, diagnostic, preanalytical variable

## Abstract

Liver disease is a major cause of morbidity and mortality worldwide. As in other fields of medicine, there is a stringent need for non-invasive markers to improve patient diagnostics, monitoring and prognostic ability in liver pathology. Cell-free circulating RNA molecules have been recently acknowledged as an important source of potential medical biomarkers. However, many aspects related to the biology of these molecules remain to be elucidated. In this review, we summarize current concepts related to the origin, transportation and possible functions of cell-free RNA. We outline current development of extracellular RNA-based biomarkers in the main forms of non-inherited liver disease: chronic viral hepatitis, hepatocellular carcinoma, non-alcoholic fatty liver, hepato-toxicity, and liver transplantation. Despite recent technological advances, the lack of standardization in the assessment of these markers makes their adoption into clinical practice difficult. We thus finally review the main factors influencing quantification of circulating RNA. These factors should be considered in the reporting and interpretation of current findings, as well as in the proper planning of future studies, to improve reliability and reproducibility of results.

## 1. Introduction

Liver disease is a significant burden for the public health system worldwide. For instance, hepatitis B virus (HBV) infection has affected approximately one third of the world’s population, and, at present, there are up to 400 million HBV surface antigen carriers worldwide [1]. There are also approximately 160 million persons chronically infected with hepatitis C virus (HCV) [2]. Both forms of chronic viral hepatitis are associated with development of liver cirrhosis and hepatocellular carcinoma (HCC). Indeed, approximately 80% of cases of hepatocellular carcinoma are associated with chronic HBV or HCV infections [3]. Liver cancer is the sixth most common cancer and the third cause of cancer-related death [4]. Due to difficulties in the management of these conditions, there is a stringent need for informative markers, that can facilitate early diagnostic, accurate prognostic and treatment monitoring in liver disease.

The presence of cell-free nucleic acids in plasma and serum has been acknowledged since the late 1940s. Later, fetal mRNA was found to be detectable in plasma of pregnant women. Specific circulating mRNA sequences have also been described in patients with cancer, cell and organ transplantation, coronary heart disease, stroke, sepsis, burns, and in several other medical fields [5]. Recent technical advances have enabled detection of hundreds of RNA sequences in the extracellular environment of healthy individuals [6]. 

In the context of an ever-increasing need for noninvasive molecular markers in medicine, circulating RNA molecules have become appealing biomarker candidates in liver disease. However, many aspects related to their origin and biological significance, remain to be clarified. Moreover, a long-acknowledged heterogeneity of technical methods employed in the assessment and interpretation of circulating RNA levels imposes a more comprehensive standardization and harmonization of biological assays. 

The aim of this review is to highlight recent advances in the study of circulating RNA molecules as biomarkers in liver pathology, and to provide a short overview on the biological properties of these molecules and the analytical challenges in their assessment.

## 2. Circulating RNA: Sources and Transportation

The presence of nucleic acids, particularly RNA, in body fluids was rather surprising, given the high amounts of nucleases in the extracellular environment. Whereas the addition of purified RNA to blood or plasma results in its immediate degradation [7], endogenous RNA is stable for several hours in plasma at room temperature. A hybridization of circulating RNA with DNA molecules was proposed as an explanation for these observations. However, the addition of RNase-H to plasma does not affect RNA recovery, thus excluding the RNA-DNA hybrid hypothesis. Instead, an association of cell-free RNA with lipids, either in the form of vesicles or lipoproteins, has been suggested. Indeed, the degradation of endogenous RNA after detergent addition to plasma samples and the retention of most of the circulating mRNA by 0.22 µm filters, support this theory [8].

### 2.1. Sources of Circulating RNA

Multiple mechanisms are involved in the release of RNA from cells. Among passive processes, RNA leakage during cellular necrosis has been cited [9]. Following cell death, RNA can reach extracellular environment bound to and protected by sub-cellular structures. RNA release via apoptotic bodies and microvesicles has been described among active processes.

Apoptosis is a highly organized process culminating with the ordered disposal of cell structures. During apoptosis, cellular RNA is packaged into granules and subsequently into apoptotic bodies, separately from the DNA [10]. Although the uptake of apoptotic bodies by neighboring cells is facilitated by exposure of phosphatidylserine on the outer leaflet of the membrane, a minority of these vesicles reaches the circulation [9].

Viable cells are also able to release microvesicles in the extracellular environment, both *in vivo* and *in vitro*. Microvesicles include a heterogeneous population of particles released as shedding vesicles and exosomes, and are now acknowledged as a constitutive part of the intercellular environment. Shedding vesicles are spherical structures with a diameter up to 200 nm, formed by the direct budding of the plasma membrane which entraps a portion of the cytosolic content. The release of shedding vesicles is mostly regulated and depends on the activation state of the source cells. Exosomes are microvesicles of 30–100 nm in diameter whose biogenesis begins with the endocytosis process, followed by the inward budding of the endosome membrane, fission and segregation of vesicles inside the multivesicular bodies. The fate of these structures via lysosomal degradation or exocytosis is dictated by specific processes. Indeed, exosomes are released both in a constitutive and a regulated fashion [11].

Cells seem to differentially release mRNA into vesicles, depending on environmental conditions [12]. It is not clear yet how certain RNA sequences are specifically enriched in membrane-derived microvesicles before secretion. Recently, Bolukbasi *et al.* [13] discovered a common pattern in the structure of several microvesicle-enriched mRNAs. A stem-loop forming sequence of 25 nt, containing a binding site for miR-1289 and a CTGCC core sequence, was common to enriched mRNAs secreted by glioblastoma cells. Also, the enrichment of a reporter mRNA into secreted microvesicles depended on miR-1289 expression in those cells.

### 2.2. Transport of Extracellular RNA Molecules

In addition to microvesicles, other forms of RNA transportation outside the cells have been described. In fact, most of miRNAs in plasma are associated with proteins [14]. The main protein transporter of plasma miRNA was identified as argonaute 2 (AGO2), the key effector protein of miRNA-mediated silencing machinery [14,15]. AGO2-bound miRNAs show remarkable stability in the extracellular space, being detectable in culture media up to two months after cell death [15]. It is thus possible that at least some of the AGO2-associated plasma miRNA originates from dead cells. Other members of the AGO family, such as AGO1, AGO3 and AGO4, with different tissue specificities, also seem to be associated with extracellular miRNA [15]. The lack of correlation between extracellular miRNAs bound to AGO1 or AGO2 proteins suggests that these miRNAs have different tissue origins [16].

MicroRNAs have a variate distribution in plasma, some of them being associated with proteins, others with exosomes, and others present in both compartments. This distribution may reflect the heterogeneity of the type and functionality of cells from which miRNAs originated. For example, the liver-specific miR-122 was detected only in protein-associated fractions, suggesting a protein carrier-related mechanism of release. On the other hand, miRNAs mainly associated with vesicles may be exported by cells adapted to vesicle secretion, such as reticulocytes and platelets [14]. However, various types of tissue injury differentially alter the abundance of miRNA molecules in circulatory compartments. For example, in alcoholic liver disease and in inflammatory liver injury, miR-122 and miR-155 are mainly associated with exosomes, whereas in drug-induced liver injury, these miRNAs predominate in the protein-rich fraction. This suggests that miRNA distribution in circulatory compartments may provide further specificity to the identification of mechanisms of liver pathology [17].

Other miRNAs, such as miR-223, are transported in plasma by high-density lipoproteins (HDL), and their delivery to recipient cells depends on scavenger receptor class B type I (SR-BI) [18]. This mechanism of transfer proved to be functional, directly altering gene expression in target cells. SR-BI-mediated transfer may serve to direct HDL-bound miRNAs into the cytoplasm and avoid their lysosomal degradation, thus increasing the chances that the message is delivered [18].

### 2.3. Circulating RNA: “Message in a Bottle”?

There is increasing evidence that RNA-carrying microvesicles produced by several cell types convey specific messages to recipient cells. *In vivo*, exosomes can be taken up by macrophages and other cells, and their RNA content is partially shuttled to the nuclei of recipient cells [19]. The active uptake of exosomes from body fluids by target cells suggests the *in vivo* relevance of exosome-mediated transfer of RNA [20].

Exosome-borne mRNA was found to be translatable [20]. Moreover, the mRNA cargo of microvesicles contains a specific subset of transcripts, rather than a random sample of the cellular RNA content [21]. The interaction between microvesicles and target cells, and the consequent transfer of genetic information, seems to be cell-specific. Thus, exosomes derived from MC/9 liver mast cells are able to transfer RNA to other mast cells, but not to CD4 cells [20]. Microvesicles derived from endothelial progenitor cells are functional both *in vivo* and *in vitro*, being able to induce an angiogenic program to human endothelial cells, via horizontal transfer of mRNA [21]. Exosomes secreted by cardiomyocytes were found to contain more than 1500 mRNA sequences. These vesicles can be taken up by fibroblasts and induce expression changes in hundreds of genes [22].

Hepatocytes can be both a source and a target for microvesicle-mediated intercellular communication. Hep3B hepatocarcinoma cells secrete microvesicles that differ in both RNA and protein content from the producing cells, and these vesicles can transmit a functional transgene to other cells. Hepatocarcinoma cells are able to reduce, via exosome-mediated transfer of miRNA, the expression of transforming growth factor beta activated kinase-1 (TAK1) in other cells. Since TAK1 is an essential inhibitor of hepatocarcinogenesis, its downregulation may promote tumor progression [23]. On the other hand, hepatoma cells can be targeted by microvesicles originating from human liver stem cells. The CD29-mediated uptake of these microvesicles by hepatoma cells results in significant inhibition of tumor cell growth and stimulation of their apoptosis both *in vitro* and *in vivo*. The anti-tumor effect of stem cell-derived microvesicles appears to depend on the horizontal delivery of a specific set of miRNAs. Since these miRNAs modulate signaling pathways differentially activated in cancer compared with normal cells, it was supposed that the effect of vesicle-dependent miRNA delivery may be specific to the functional state of the target cell rather than the gene expression of source cells [24]. Besides miRNA, other small RNA species can be exchanged between cells in a contact-independent manner. As a possible therapeutic application, the ability of intercellular exchange of genetic material to interfere with viral replication was tested in a HCV infection model [25]. Human and mouse liver cells, as well as primary human B lymphocytes, appeared able to deliver small silencing RNA targeting the HCV genome and the HCV receptor CD81. This transmission of siRNA was partially mediated by exosomes [25].

Although RNA-mediated non-contact intercellular communication is an exciting emerging concept, many aspects related to the physiopathological conditions leading to release of RNA from cells and the signaling properties of these molecules are still to be described.

## 3. Circulating RNA as Biomarkers of Liver Injury

Ideally, circulating biomarkers of tissue injury should be expressed at high levels preferentially or exclusively in the tissue of interest. They should also have low circulating levels in healthy individuals, whereas important changes (usually increases) in their blood concentrations should be detectable upon tissue injury [26]. Additionally, they should allow rapid, accurate and inexpensive detection, be invariant to unrelated conditions and easily translatable from pre-clinical to clinical observations. Tissue-specific transcripts possess several of these requisites [27]. Although rigorous validation is still necessary, circulating RNAs appear as a promising source of biomarkers in various forms of liver disease (Table 1).

### 3.1. Liver Toxicity

Hepatocyte-specific RNA sequences including miR-122, albumin (ALB), microglobulin/bikunin precursor, haptoglobin (HP), fibrinogen B β-polypeptide, apolipoprotein H and vitamin D binding protein, were assessed as potential biomarkers in various animal models of liver toxicity [9,26,28,29,30]. Several classes of hepatotoxic compounds were tested, including CCl_4_ and CBrCl_3_, d-galactosamine, and acetaminophen. A common finding of these studies is the increase of circulating liver-derived RNAs in treated animals. Peak levels of specific RNAs are correlated with classical liver injury markers, such as serum transaminase activities. Circulating RNAs rise in a time and dose-dependent manner in response to liver injury. They also constitute a more sensitive marker of tissue damage, and can be detected earlier than changes in alanine aminotransferase (ALT) levels, even before the onset of observable histological modifications. These circulating RNAs are also more specific than serum transaminases, since their levels are not influenced by injury affecting other tissues, including skeletal muscle and brain.

**Table 1:** Circulating RNA as biomarkers in liver disease.

Marker	Condition	Change	Diagnostic Performance	Validation in Independent Cohort	Reference
*Liver toxicity*					
ALB, AMBP	Liver toxicity (DGAL, APAP) *	up	Not reported	N/A	[28]
ALB, AMBP, APOH, GC	Liver toxicity (various compounds) *	up	Not reported	N/A	[29]
ALB, FGB, HP	Liver toxicity (DGAL, APAP) *	up	Not reported	N/A	[9]
miR-122	Liver toxicity (CBrCl_3_, CCl_4_) *	up	Not reported	N/A	[26]
miR-122, miR-192	Liver toxicity (APAP) *	up	Not reported	N/A	[30]
miR-122, miR-192	Liver toxicity (APAP)	up	Not reported	no	[31]
*Liver pathology (general)*					
ALB	HCC, liver cirrhosis, active CHB *vs.* controls	up	Sensitivity 85.5%, specificity: 92.8% for liver pathology	no	[32]
miR-885-5p	HCC, CHB and liver cirrhosis *vs.* control	up	AUC: 0.904, sensitivity 90.53%, specificity: 79.17% for liver pathology	yes	[33]
miR-1225-5p, -1275, -638, -762, -320c, -451, -1974, -630, -1207-5p, -720, -1246 and -486‑5p	CHC, CHB, NASH, and controls	-	Accuracy of distinction among conditions: 87.5%	yes	[34]
*Chronic hepatitis C*					
miR-92a and miR-423	CHC *vs.* control	up	AUC: 0.996; sensitivity: 97.9%; specificity: 99.4%	yes	[35]
miR-1225-5p, -1275, -638, -762, -320c, -451, -1974, -1207-5p and -1246	CHC *vs.* control	-	Diagnostic accuracy: 96.6%	yes	[34]
miR-122, -16, -34a	CHC *vs.* control	up	Not reported	yes	[36]
miR-122, miR-16	Early CHC (F0-F1) *vs.* control	-	AUC: 0.90 and 0.92, respectively	no	[36]
miR-483-5p, miR-671-5p	Liver fibrosis in CHC patients	up; correlation	Accuracy 87.5%, OR 14.25 (F0 *vs.* F1-F3)	no	[34]
let-7a, miR-106b, -1274a, -130b, -140-3p, -151-3p, -181a, -19b, -21, -24, -375, -548l, -93 and -941	Liver fibrosis in CHC patients	down; correlation		no	[34]
miR-571	Liver cirrhosis in CHC patients	up	AUC: 0.91 for the presence of cirrhosis	no	[37]
miR-122	Liver fibrosis in CHC patients	no correlation	Not reported	no	[38,39]
miR-122, miR-34a	Liver fibrosis and activity in CHC patients	up; correlation	Not reported	no	[36]
miR-122	Necroinflammatory activity in CHC patients	up; correlation	Not reported	no	[38]
miR-1914 *, -193a-5p, -22, -659 and -711	Liver inflammation in CHC patients	up; correlation	Accuracy for A1, A2, and A3: 71.9%, 75% and 82.8%, respectively	no	[34]
miR-1274b, -197, -1974, -21, -34a,-451, -548d-5p, -760 and -767-3p	Liver inflammation in CHC patients	down; correlation		no	[34]
*Chronic hepatitis B*					
miR-375, -92a, -10a, -223, -423, -23b/a, -342-3p, -99a, -122a, -125b, -150 and let-7c	CHB *vs.* control	up	Not reported	yes	[35]
miR-375, -10a, -223 and -423	CHB *vs.* control	up	AUC: 0.999; sensitivity: 99.3%; specificity: 98.8%	yes	[35]
miR-122	CHB *vs.* control	up	AUC: 0.989	yes	[40]
miR-122	CHB (active) *vs.* control	up	AUC: 0.762	no	[41]
ALB, APOA2, HP, CYP2E1	CHB (active) *vs.* control	up	AUC: 0.945, 0.909, 0.834, 0.801, respectively	no	[41]
miR-122, -638, -575, -572 and -744	CHB *vs.* control	-	CHB *vs.* healthy: AUC: 0.98, 1.00, 0.91, 0.95 and 0.95, respectively	no	[42]
miR-21, -122 and -223	CHB *vs.* control	up	CHB *vs.* healthy: AUC: 0.91, 0.93, and 0.88, respectively	no	[43]
miR-99a, -100, -122, -122 *, -125b, -192, -192 *, -193b, -194, -215, -365, -455-5p, -455-3p, -483-3p, -885-5p and -1247	CHB: HBeAg positive, HBeAg negative and healthy children	up; HBeAg positive > HBeAg negative > healthy	Not reported	no	[44]
miR-99a-5p, -100-5p, -122-5p, -122-3p, -125b-5p, 192-5p, -192-3p, -193b-3p, -194-5p, -215, -365a-3p, -455-5p, -483-3p and -855-5p	CHB: immunological phases of HBV infection in children	down; immune-tolerant > immune-active > immune-inactive	Not reported	no	[45]
miR-10a and miR-125b	CHB *vs.* HBV-positive HCC	up	AUC: 0.992; sensitivity: 98.5%; specificity: 98.5	yes	[35]
*Hepatocellular carcinoma*					
ALB	HCC	up	AUC: 0.72, sensitivity 73%, specificity 70% for prediction of 2-year HCC recurrence	no	[46]
MiR-21 + AFP (protein)	HCC	up	HCC *vs.* chronic hepatitis: AUC: 0.773, sensitivity 61.1%, specificity 83.3%. HCC *vs.* healthy: AUC: 0.953, sensitivity 87.3%, specificity 92.0%.	no	[47]
miR-122	HCC *vs.* control	up	AUC: 0.869, sensitivity 81.6%, specificity 83.3%	yes	[48]
miR-222, miR-223	HCC *vs.* control	up	Not reported	yes	[48]
miR-21	HCC *vs.* control	down	Not reported	yes	[48]
miR-375	HCC *vs.* control	up	AUROC: 0.96, specificity: 96%; sensitivity: 100%	yes	[35]
miR-375, -25 and let-7f	HCC *vs.* control	up	AUC: 0.997; sensitivity: 97.9%; specificity: 99.1%	yes	[35]
miR-23b, -423, -375, -23a and -342-3p	HBV-positive HCC *vs.* control	up	AUC: 0.999; sensitivity: 96.9%; specificity: 99.4%	yes	[35]
miR-21, -122 and -223	HCC *vs.* control	up	AUC: 0.87, 0.79, and 0.86, respectively.	yes	[43]
miR-122, -192, -21, -223, -26a, -27a, and -801	HCC	-	AUC: 0.888; 0.941 (*vs.* healthy); 0.842 (*vs.* CHB); 0.884 (*vs.* cirrhosis)	yes	[49]
*Liver transplantation*					
ALB	Liver transplant complications	up	Not reported	no	[50]
miR-122 and miR-148a	post-transplantation liver injury	up	Not reported	no	[51]
*Non-alcoholic fatty liver disease*					
miR-122, miR-16	Early NAFLD (simple steatosis) *vs.* control	up	AUC: 0.93 and 0.96, respectively	no	[36]
miR-122, -638, -575, -572 and -744	NASH *vs.* control	-	AUC: 0.80, 0.97, 0.90, 0.85, and 0.96, respectively.	no	[42]
miR-122, -192, -19a, -19b, -125b and -375	NAFLD *vs.* control	up	AUC: ~0.7 (for miR-122, -192 and -375)	yes	[52]
miR-21, -34a, -122 and -451	NAFLD *vs.* control	up	Not reported	no	[53]
miR-122-5p, -1290, -27b-3p and -192-5p	NAFLD *vs.* control	up	AUC: 0.856, sensitivity 85.55%, specificity 73.3%	yes	[54]

* animal model; APAP, acetaminophen; AUC, area under the receiving operator characteristic (ROC) curve; CHB, chronic hepatitis B; CHC, chronic hepatitis C; DGAL, d-galactosamine; HCC, hepatocellular carcinoma; NAFLD, non-alcoholic fatty liver disease; NASH, non-alcoholic seatohepatitis.

Interestingly, liver-derived mRNAs and the reference transcripts are also present in plasma vesicles from non-treated animals, suggesting a physiological, active secretion of microvesicles, independent of tissue injury. In the case of treated animals, liver mRNAs are present in both microvesicles and cellular debris isolated from plasma. Their circulating levels are elevated despite a reduced expression in liver tissue. This suggests a shift in the secretion mechanism from an active one, in the absence of injury, to both active and passive, at cytotoxic doses of chemicals [9].

Similar changes were reported in humans. In patients with acetaminophen poisoning, serum miR-122 was significantly correlated with peak serum ALT activity. Also, miR-122 levels returned to baseline earlier than ALT, suggesting a shorter circulatory half-life of the microRNA [31]. Another study identified 11 circulating miRNAs, including miR-122, that were able to distinguish patients with acetaminophen poisoning from healthy controls and patients with ischemic hepatitis, another form of severe liver injury [55]. The levels of these miRNAs in plasma and serum increased earlier than ALT after acetaminophen overdosage, and also returned to normal more rapidly than ALT, in response to N-acetyl cysteine therapy, suggesting their potential use in treatment monitoring in these patients.

### 3.2. Chronic Viral Hepatitis

HBV infection is another condition associated with altered levels of plasma miRNAs. Thirteen circulating miRNAs, including miR-375, miR-92a, miR-10a, miR-223, miR-423, miR-23b/a, miR-342-3p, miR-99a, miR-122a, miR-125b, miR-150, and let-7c, were found to be upregulated in chronic HBV carriers compared with healthy controls [35]. This panel could separate HBV cases from controls and HCV carriers, and also HBV-positive HCC from controls, HBV cases, and HCV cases [35]. Four other serum miRNAs (miR-572, miR-575, miR-638 and miR-744), together with miR-122 were reported to distinguish chronic hepatitis B (CHB), non-alcoholic steatohepatitis (NASH) and control individuals [42]. Similarly to CHC, the increase of miR-122 in HBV infection positively correlates with plasma ALT [40,41]. However, increases in plasma miR-122 coincide with histologic alterations in the same individuals, even in cases with normal levels of ALT. Plasma miR-122 seems to be superior to ALT in the accuracy of detecting HBV-induced liver damage [40]. 

Serum HBeAg is regarded as a surrogate marker for active viral replication in CHB patients. Interestingly, the replication status of HBV translates into different circulating miRNA profiles [44]. A panel of 16 miRNAs, related to signaling or cancer pathways, had different expression patterns in plasma of HBeAg positive, HBeAg negative and healthy children. A strong correlation was observed between the circulating levels of these miRNAs and HBV DNA [44]. Concentrations of several plasma miRNAs also seem to change among the immunological phases of HBV infection in children [45]. Four circulating miRNAs, namely miR-99a-5p, -122-5p, -122-3p and -125b-5p, decreased in immune-tolerant and immune-active children, whereas their levels were stable in immune-inactive children [45]. Among these miRNAs, miR-122 negatively regulates HBV gene expression and replication by binding to highly conserved regions of the viral polymerase and the 3' untranslated region (UTR) of the core protein mRNA [56].

Interestingly, several plasma miRNAs correlate with HBs antigenemia in HBV-infected persons [45]. Zhang *et al.* [41] found miR-122 to correlate more with HBsAg titre than with serum ALT. These observations are consistent with the earlier finding that HBsAg particles carry hepatocellular miRNAs and the associated AGO2 protein [57]. Apparently, liver-specific mRNA sequences can also be entrapped along with miRNAs, during the process of HBs particle assembly and secretion [41].

In chronic hepatitis C (CHC), serum miRNAs correlate with the stage of liver injury. miR-571 is upregulated in the serum of CHC patients with liver cirrhosis, reflecting a concordant regulation in the liver tissue in response to the pro-fibrogenic cytokine transforming growth factor beta (TGF-β) [37]. Similarly, a good correlation in gene transcription was observed between liver and serum in CHC patients. This correlation involved a variety of transcripts, including genes for immune markers, or related to inflammation, apoptosis and matrix turnover [58].

Serum miRNA profile partially correlates with liver fibrosis stage and inflammation grade in CHC patients. One study reported a high accuracy of discrimination between fibrosis stage F0 and F1–F3 in CHC patients, through assessment of serum miRNAs. The same set of miRNAs could also separate CHC from CHB and NASH [34].

miR-122 was reported as a positive regulator of HCV replication and particle production [59,60]. miR-122 promotes HCV replication in experimental models, possibly by facilitating the folding of viral RNA and its sequestration in active replication sites [61]. miR-122 also activates HCV translation by binding to its 5' UTR, in a process mediated by AGO proteins [62]. However, HCV viral load is not correlated with liver miR-122 expression in infected patients [63], and plasma miR-122 does not correlate with HCV viral load either [36]. Instead, circulating miR-122 correlates with serum transaminases and with necroinflammatory activity in CHC, whereas intrahepatic miR-122 shows a negative correlation with the extent of liver damage [38]. The lack of parallelism between cellular and circulating miR-122 may be explained by an increased release of miRNA from affected cells, despite the downregulation of its expression. 

There are conflicting reports regarding the relationship between circulating miR-122 and liver fibrosis. In one study investigating approximately 50 patients, plasma miR-122, as well as miR-34a, correlated with liver fibrosis severity, and was suggested as a suitable marker [36]. In contrast, two other studies including 68 and 164 patients respectively, did not find a significant correlation between circulating miR-122 and fibrosis [38,39]. Unfortunately, the lack of details regarding the technical assessment of miRNAs in these studies, especially the preanalytical variables, makes the reported results difficult to interpret and compare. As discussed below, a simple factor such as sample storage time at room temperature before centrifugation, can bias the results and should be accounted for [64].

### 3.3. Hepatocellular Carcinoma

MicroRNAs play key roles in the development of hepatocellular carcinoma, by interfering with crucial cancer-associated pathways [65]. miR-21 is highly overexpressed in HCC tissue and contributes to tumor growth and spread by modulating the expression of phosphatase and tensin homolog (PTEN) tumor suppressor [66]. Inhibition of miR-21 in cultured HCC cells impairs tumor cell proliferation, migration and invasion, whereas enhanced expression of miR-21 has the opposite effect [66]. Upregulation of miR-222 and downregulation of miR-223 were observed in primary HCC as compared with adjacent normal liver tissue [67]. Restoration of miR-223 expression severely impairs viability of HCC cell lines by targeting Stathmin 1 [67]. miR-122 is a powerful tumor suppressor, by inhibiting angiogenesis, tumorigenesis and HCC intrahepatic metastasis, partially by targeting ADAM metallopeptidase domain 17 (ADAM17) [68]. Accordingly, miR-122 is strongly downregulated in HCC [68]. 

Interestingly, modified circulating levels of these RNAs were repeatedly found in HCC patients. Plasma miR-21 was higher in HCC patients than in chronic hepatitis and healthy volunteers, and its levels correlated with expression in tumor tissue [47]. Its diagnostic value was better than that of alpha fetoprotein (AFP), and improved for the combination of the two markers. Diagnostic sensitivity, specificity and accuracy of the combination of miR-21 and AFP for the diagnosis of HCC were higher than 90%, suggesting that circulating RNAs are a useful addition to classical tumor markers. 

Chronic HBV infection is a major cause of HCC. By comparison of circulating miRNA profiles in healthy subjects and in patients with various stages of HBV infection, seven miRNAs were identified as potential markers of HCC [49]. The panel, comprising miR-122, miR-192, miR-21, miR-223, miR-26a, miR-27a, and miR-801, was developed on large groups of participants (407 and 390 participants in the training and validation groups, respectively) and demonstrated a high diagnostic accuracy for HCC, with an area under the receiving operator characteristic curve (AUC) of 0.888 in the validation set. The 7 miRNA panel could also differentiate HCC from healthy (AUC 0.941), chronic hepatitis B (AUC 0.842), and cirrhosis (AUC 0.884), respectively. Among the seven miRNAs of the panel, tumor expression of miR-26a and miR-192 was associated with early recurrence of HCC [69]. In an independent study, Xu *et al.* [43] showed that miR-21, miR-122, and miR-223 had higher serum levels in HCC and CHB patients than in healthy controls. Serum levels of two miRNAs, miR-10a and miR-125b, were shown to be lower in HBV-positive HCC than in CHB patients, and the combination of the two markers could accurately distinguish between these pathological conditions [35]. Accordingly, miR-125b is underexpressed in HCC tissue and was recently identified as a potential tumor suppressor [70].

A set of three miRNAs, miR-375, miR-25 and let-7f could separate HCC from healthy subjects with a sensitivity and specificity of 97.9% and 99.1%, respectively [35]. When considered alone, miR-375 had 100% sensitivity and 96% specificity in predicting HCC [35].

Gui *et al.* [33] identified a single serum miRNA that could differentiate patients with liver pathologies from healthy controls with more than 90% sensitivity and 79% specificity. miR-885-5p was significantly more abundant in serum from patients with HCC, CHB and liver cirrhosis. 

miRNAs are not the only circulating RNA molecules deregulated in patients with liver cancer. A study reported that more than 90% of the investigated HCC patients had increased plasma ALB mRNA, whereas less than 50% had a high level of circulating AFP mRNA [32].

Besides HCC, higher levels of plasma ALB mRNA were found in cirrhosis and active (but not inactive) hepatitis B patients than in healthy controls. Plasma ALB mRNA had a diagnostic sensitivity of more than 85% and a specificity over 90% for the detection of these pathologies [32]. It was debated whether the detected ALB mRNA originated from hepatocytes or from an illegitimate transcription process in other cells. It is known that peripheral blood mononuclear cells transcribe ALB in almost one third of healthy individuals and in about 90% of patients with chronic hepatitis [71]. Recently, it was confirmed that ALB mRNA detected in whole blood comprised a mixture of molecules released by blood cells and hepatocytes. However, using a RNA single nucleotide polymorphism approach to genotype ALB mRNA in the plasma of liver and bone marrow transplantation recipients, it was shown that cell-free ALB transcripts are only of hepatic origin [32].

### 3.4. Non-Alcoholic Fatty Liver Disease

Non-alcoholic fatty liver disease (NAFLD) is the most common cause of chronic liver disease in America [72], and includes a spectrum of histological hepatic changes ranging from simple steatosis to NASH. Depending on their severity, these pathological changes alter the expression of several hepatic miRNAs. Of the 474 investigated miRNAs, Cheung *et al.* [73] found 46 that were differentially expressed in the liver of NASH patients compared with controls with normal liver histology. Hepatic miR-122 is significantly downregulated in NASH. Accordingly, liver mRNA and protein levels of sterol response element binding protein 1c (SREBP-1c), fatty acid synthase, and 3-hydroxy-3-methylglutaryl-coenzyme A reductase, some of the miR-122 targets involved in lipid biosynthesis, are increased in NASH patients [73]. A decrease of liver miR-122 levels in NASH patients was also confirmed by *in situ* hybridization, in an independent study [52]. Interestingly, miR-122 is preferentially localized in lipid-laden hepatocytes, near the cell membrane, as if ready to be secreted. Accordingly, circulating miR-122 levels are more than 7-fold increased in NASH patients compared with controls [52]. Serum miR-122 correlates with ALT levels and liver fibrosis in NAFLD patients. It also performs slightly better in the diagnosis of NAFLD severity than classical liver disease markers, including aspartate aminotransferase (AST), ALT and plasma caspase generated cytokeratin-18 fragments. Besides miR-122, Pirola *et al.* [52] found two other miRNAs, miR-192 (upregulated by TGFβ1) and miR-375 (a key regulator of glucose homeostasis), with lower liver expression and higher serum levels in NASH compared with simple steatosis. These changes were subsequently validated in an independent cohort. Similarly, Yamada *et al.* [53] found increased levels of serum miR-21, miR-34a, miR-122 and miR-451 in participants with NAFLD from a large group of non-selected Japanese people attending health examinations. Among these markers, only miR-122 correlated with the severity of liver steatosis.

A panel with high diagnostic accuracy for NAFLD was recently described [54]. The panel, consisting of four miRNAs (miR-122-5p, miR-1290, miR-27b-3p and miR-192-5p), was developed and validated on two large independent cohorts and seems to have better sensitivity and specificity for NAFLD than ALT and the non-invasive score FIB-4. Moreover, the performance of the miRNA panel was not influenced by disease severity. Of note, miR-27b, a member of this panel, promotes fat accumulation in hepatocytes via repression of peroxisome proliferator-activated receptor-α and angiopoietin-like protein 3 [74], and was previously found upregulated in the liver of NASH patients [73].

### 3.5. Liver Transplantation

In liver transplant recipients, serial assessment of circulating ALB mRNA can be used to detect the progression of hepatic complications [50]. Hepatic injury and rejection after liver transplantation can also be monitored using hepatic-specific miRNAs [51]. Accordingly, serum miR-122 and miR-148a were elevated in patients with post-transplantation liver injury and correlated with serum transaminases. During episodes of acute rejection, their levels increased up to 20-fold, and their peak occurred earlier than in the case of transaminases [51]. It was also speculated that liver-derived mRNA levels could be used as an indicator of the quality of transplanted grafts or as an aid in the choice of proper immunosuppresive therapy in transplant recipients. However, further research is needed for the proper validation of circulating mRNA as biomarkers in this field [75].

### 3.6. Prognostic Value of Circulating RNAs in Liver Disease

Although the diagnostic value of circulating RNAs in liver disease has been assessed in many comparative studies, their prognostic relevance in longitudinal designs was addressed to a much lower extent. 

The prognostic value of serum miR-122 in advanced liver disease was recently evaluated. Waidmann *et al.* [76] observed that patients with hepatic decompensation had lower circulating miR-122 than those with compensated liver disease. In their cohort, low serum miR-122 was associated with complications such as ascites, spontaneous bacterial peritonitis and hepatorenal syndrome in cirrhotic patients. Multivariate Cox analysis revealed that miR-122 was an independent predictive factor for survival in patients with cirrhosis. The authors validated their observations in a second independent cohort. However, the threshold for serum miR-122 was chosen relative to their study group, dividing the cohorts into the third of patients with highest serum miR-122 levels and the other two thirds with lower miR-122. Since the general recommendation in clinical chemistry practice is that each laboratory should establish its own reference ranges for the analytes it assesses, such a threshold choice is acceptable. However, the lack of a quantitative value (e.g., in copies/microliter) makes their results difficult to translate into other laboratories. High serum miR-122, as well as high miR-1, also predicts longer overall survival in HCC patients [77]. 

Plasma ALB mRNA is detectable in the majority of HCC patients [46]. Preoperative levels of plasma ALB mRNA predict survival and HCC recurrence. The predictive ability of the transcript is higher than the size and number of tumors (the usual preoperative radiological criteria) and is similar to the presence of vascular invasion (pathological criterion). Cheung *et al.* [46] detected ALB mRNA in almost all HCC, in contrast with AFP mRNA, which was only detectable in some of the cases.

## 4. Pre-Analytical and Analytical Challenges in the Assessment of Circulating RNA

Real-time PCR is the preferred format for the quantification of cell-free circulating RNA. Despite the high sensitivity and specificity attainable in RNA quantification with the available technology, conflicting results have been reported in different studies on circulating RNA as biomarkers. It was rapidly observed that a multitude of factors were able to influence circulating RNA quantification at both pre-analytical and analytical levels. In consequence, several studies were performed to assess the pre-analytical variables affecting the quantification of circulating RNA, especially miRNA. In contrast, mRNAs were less studied from this point of view.

Low level haemolysis often occurs during collection of blood samples. This phenomenon can increase the levels of red blood cell-related miRNAs in plasma, thus affecting any potential miRNA biomarker that is also present within erythrocytes [78]. miR-16, which is expressed at high levels in circulating cells, is greatly affected by haemolysis [78,79]. Other blood cells can also serve as a source of circulating miRNAs. Variations in blood cell counts and haemolysis can alter plasma miRNA levels by up to 50-fold [80]. It is not clear yet to what extent haemolysis affects other extracellular RNA species. RNAs not expressed in blood cells, such as miR-122, are not increased upon sample haemolysis [64]. Short noncoding RNA and mRNA are less stable in human plasma than miRNA [15] and thus, one can expect their levels to be less influenced by haemolysis. However, haemolysis entrains a release of RNase inhibitors from lysed cells, thus reducing degradation of certain circulating RNAs, such as miR-122 [64]. The general recommendation is that free hemoglobin in samples be assessed by spectrophotometry and haemolyzed samples be removed from analysis, unless it is rigorously demonstrated that the RNA sequences of interest, including reference genes, are not affected by haemolysis [78,79]. Alternatively, if only extracted material is available, putative haemolyzed samples can be identified by the relative expression of the erythrocyte-specific miR-451 and the stable miR-23a [81].

The choice of sample type also influences results. Although serum and plasma levels of several miRNAs, including miR-15b, miR-16, and miR-24, are strongly correlated [82], these molecules are present at higher concentrations in plasma than in serum [79]. Differences between serum and plasma miRNA profiles showed a certain association with miRNA from platelets, suggesting that coagulation may distort extracellular miRNA concentrations in samples [83]. In the case of plasma, miRNA quantification is affected by the type of anticoagulant. For example, the fluoride/oxalate mixture enables higher sensitivity in miRNA detection than citrate and ethylenediaminetetraacetic acid (EDTA) [84]. 

Sample pre-processing steps affect subsequent quantification results. Most of extracellular RNA is removed by filtration through 0.2 µm filters [8]. Also, total RNA content of plasma samples decreases as higher centrifugation forces are applied [85]. Exosome enrichment can improve detection of certain RNA targets, but can significantly distort expression profiles compared with plasma. This can be due to the different concentrations of targets in exosomes *vs.* exosome-free fluids [86] and to the unbalanced distribution of various RNA sequences inside and outside microvesicles [14]. 

Delays in sample processing seem to decrease the amounts of detectable mRNA [87] and miRNA [88] in plasma. Serum storage at room temperature biases miRNA profiles, increasing the proportion of vesicle-associated miRNAs as compared to protein-associated miRNAs [64]. It seems that microvesicle-borne miRNAs are more resistant to RNase activity than are miRNAs complexed with proteins. Relative expression profiles of certain plasma miRNAs were reportedly unaffected by transportation times of up to 48 h prior to centrifugation, as compared to samples processed within four hours of collection [44,45]. However, in order to limit pre-analytical variability, sample storage at room temperature should be minimized, and a temperature of −70 °C or below is recommended for long-term storage of plasma [89]. Although a certain loss of miRNAs can be observed, successful quantification is possible in samples stored for up to 12 years at −80 °C, suggesting the utility of long-term archived samples in retrospective studies [88]. It was found that a single freeze-thaw cycle does not significantly degrade plasma mRNA [7]. However, in a direct comparison, serum miRNAs were found to be more stable than high molecular weight RNAs (including GAPDH and β-actin mRNAs) when samples were subjected to multiple freeze-thaw cycles [90]. 

The yield and reproducibility of RNA extraction from body fluids are method-dependent. The column-based mirVana PARIS kit was repeatedly reported among the best performing kits for the isolation of circulating miRNA, giving the highest yields of purified nucleic acids [79,89]. McDonald *et al.* [79] found that kits not employing a phenol-chloroform isolation step, such as Roche High Pure miRNA Isolation Kit, performed better in terms of reproducibility. There are conflicting reports regarding miRNA isolation using liquid-liquid extraction methods. Whereas some researchers found TRIzol-LS extraction equal or better than column-based kits for serum miRNAs [79], others arrived at the opposite conclusion [89]. Plasma RNA recovery and detection do not necessarily parallel the sample input volume, as several blood-borne inhibitors may co-purify with nucleic acids. The addition of small amounts of inhibitor-resistant polymerase to common real-time PCR reaction mixes seems to improve detection of circulating miRNAs [84]. Storage time of purified nucleic acids in elution buffer prior to assessment by real-time PCR may also be an important factor, since long-term stability of RNA may depend on the nature of the storage solution. Thus, some researchers found that long-time storage of purified miRNAs isolated from clinical samples increased quantification variability compared with long-time storage of unextracted samples [79]. Conversely, others reported very good stability of purified miRNAs during prolonged storage [89]. 

Finally, target detection and normalization methods fundamentally contribute to quantification results. In a direct comparison of real-time PCR low-density arrays for plasma miRNA quantification, locked nucleic acid-based miRCURY platform performed better than the TaqMan counterpart in the case of low expression targets. At higher concentrations of miRNA, the performance of the two platforms was similar [91]. 

It is important that all these technical details be considered and kept as constant as possible within a study, in order to improve the consistency of the results and minimize the risk of bias.

Many studies on miRNA expression utilize U6, RNU6B or 18S as internal normalizers. However, this approach is controversial, since these targets are not miRNAs, thus their origin, stability, and efficiency of their extraction, reverse transcription and amplification may be different from those of miRNAs. If the reference gene method is used for normalization of miRNA expression data, reference miRNAs should be used instead. For example, the combination of miR-26a, miR-221 and miR-22*, was found as the most stable set of reference genes for serum miRNA assessment in HBV carriers *vs.* healthy controls [92]. 

Reference targets should be properly validated in each study, since they may be influenced by several physiological or demographic conditions affecting the studied groups of patients [93]. A number of miRNAs were shown to have gender-related circulating concentrations. Thus, plasma miR-130b and miR-18b are higher in males [83], whereas miR-548-3p, miR-1323, miR-940 and miR-1292 are upregulated in females [94]. Pathophysiological changes in sample matrix, related to the studied condition or disease may also entrain changes in the levels of detection inhibitors, and thus become a source of bias. 

Due to the multiple variables affecting the results, reports on circulating RNAs should include relevant details regarding the analytical workflow [95,96]. Guidelines for reporting results of real-time PCR experiments have been issued [97] and constitute a useful aid in the standardization of reports regarding development of biomarkers based on circulating nucleic acids. Such a standardization would allow more accurate comparisons of data obtained in different laboratories and lead to a better understanding of results. 

## 5. Conclusions and Perspectives

Despite a wealth of evidence for the presence of RNA molecules in body fluids, the origin and, more importantly, the function of these RNAs in the extracellular environment remains poorly understood. An intriguing possibility is that these molecules are related to the process of intercellular communication. Given that certain RNAs are selectively expressed in specific organs, it may be possible to monitor the physiopathological conditions of organs by the levels of circulating organ-specific RNAs. This possibility opens up new perspectives in the development of non-invasive markers of liver disease. 

Finding of informative biomarkers is not only critical for understanding of disease-related physiopathological processes, but is also important for therapeutic development. Current technology for the assessment of circulating RNAs in liver disease has several advantages over the detection of protein-based markers, including higher sensitivity and wider dynamic range, and its use has already led to very encouraging results. Undoubtedly, extracellular RNAs will constitute an excellent addition to classical noninvasive markers of liver pathology. However, standardization of sample preparation, quality assessment and quantification of circulating RNA molecules is urgently needed. It is also a key prerequisite for the adoption of cell-free RNA markers into clinical practice.
